# Recapitulation of HIV-1 Neutralization Breadth in Plasma by the Combination of Two Broadly Neutralizing Antibodies from Different Lineages in the Same SHIV-Infected Rhesus Macaque

**DOI:** 10.3390/ijms25137200

**Published:** 2024-06-29

**Authors:** Yanxin Gai, Nan Gao, Zhaoyang Mou, Chumeng Yang, Libian Wang, Wanshan Ji, Tiejun Gu, Bin Yu, Chu Wang, Xianghui Yu, Feng Gao

**Affiliations:** 1 National Engineering Laboratory for AIDS Vaccine, School of Life Sciences, Jilin University, Changchun 130012, China; gaiyx20@mails.jlu.edu.cn (Y.G.); gaonan15@mails.jlu.edu.cn (N.G.); mouzy22@mails.jlu.edu.cn (Z.M.); yangcm1319@mails.jlu.edu.cn (C.Y.); lbwang22@mails.jlu.edu.cn (L.W.); jiws21@mails.jlu.edu.cn (W.J.); gutj@jlu.edu.cn (T.G.); yubin@jlu.edu.cn (B.Y.); wangchu18@jlu.edu.cn (C.W.); 2 Key Laboratory for Molecular Enzymology and Engineering, The Ministry of Education, School of Life Sciences, Jilin University, Changchun 130012, China; 3 Institute of Molecular and Medical Virology, School of Medicine, Jinan University, Guangzhou 510632, China; 4 Key Laboratory of Viral Pathogenesis & Infection Prevention and Control, Jinan University, Ministry of Education, Guangzhou 510632, China

**Keywords:** neutralization, rhesus macaque, vaccine, HIV-1, simian-human immunodeficiency virus, broadly neutralizing antibodies

## Abstract

Viral infection generally induces polyclonal neutralizing antibody responses. However, how many lineages of antibody responses can fully represent the neutralization activities in sera has not been well studied. Using the newly designed stable HIV-1 Env trimer as hook, we isolated two distinct broadly neutralizing antibodies (bnAbs) from Chinese rhesus macaques infected with SHIV_1157ipd3N4_ for 5 years. One lineage of neutralizing antibodies (JT15 and JT16) targeted the V2-apex in the Env trimers, similar to the J038 lineage bnAbs identified in our previous study. The other lineage neutralizing antibody (JT18) targeted the V3 crown region in the Env, which strongly competed with human 447-52D. Each lineage antibody neutralized a different set of viruses. Interestingly, when the two neutralizing antibodies from different lineages isolated from the same macaque were combined, the mixture had a neutralization breath very similar to that from the cognate sera. Our study demonstrated that a minimum of two different neutralizing antibodies can fully recapitulate the serum neutralization breadth. This observation can have important implications in AIDS vaccine design.

## 1. Introduction

Human immunodeficiency virus (HIV) infection is a huge threat to global health and has claimed over 40 million lives so far, yet the pandemic continues to spread in the world [1]. Currently, there are still no effective vaccines in sight to prevent this devastating infectious disease. One of the major reasons is the extraordinarily high level of genetic variation which demands cross-reactive immune responses induced by any vaccines [2]. Studies show that about 20% of HIV-1-infected individuals who are infected for 2 to 4 years have developed cross-neutralizing activity [3,4,5,6,7]. Sera from such individuals can neutralize a wide range of HIV-1 strains by recognizing conserved regions of the HIV-1 envelope glycoprotein (Env) [8,9,10]. The main epitopes of the broadly neutralizing antibodies (bnAbs) are the V1V2 region at the Env apex, the CD4 binding site (CD4bs), the glycosylated V3 region, the silent face of the gp120, the gp120-gp41 interface, the fusion peptide region, and the membrane-proximal external region on gp41 [11,12]. It will be key to develop HIV-1 vaccines that can elicit potent bnAbs, and this has been one of the major focuses for the development of neutralizing antibody-based acquired immunodeficiency syndrome (AIDS) vaccines [13,14,15,16]. However, no vaccine candidates can induce bnAbs yet [17,18,19,20]. Some studies show that polyclonal antibody responses targeting distinct epitopes together can also achieve broad neutralization [21,22,23,24,25,26,27,28,29,30,31]. One of these studies described four different epitope nAb lineages from the same individual, which target CD4bs, the silent face, the gp120-gp41 interface, and the V3 epitope on HIV-1 Env. When these different nAb lineages are combined, they can achieve considerable neutralization breadth [27]. This indicates that compared to bnAbs targeting one epitope, the combination of neutralizing antibodies targeting different epitopes can significantly increase the neutralization breadth. Thus, it is possible for an HIV vaccine to achieve broad neutralization through targeting multiple Env epitopes. A vaccine with multiple bnAbs elicited will be much more likely to protect against infection by the majority of viruses [32,33].

Non-human primates (NHPs), with high genetic similarity to humans, are important animal models for AIDS vaccine research [34,35,36]. HIV-1 initially originated in chimpanzees [37], but it failed to effectively infect NHPs after it was adopted in humans. To evaluate the protection efficacy and ability to elicit bnAbs through vaccination, a mosaic simian-human immunodeficiency virus (SHIV) between HIV and SIV which infects monkeys has been generated by replacing the *env* gene in the SIV genome with that from HIV-1 [34]. Most of the studies with SHIV-infected rhesus macaques found that the potent neutralizing antibodies often target the V2 apex or V3 regions [38,39,40,41,42,43,44]. Previously, we found that nAbs with multiple specificities could be elicited in SHIV_1157ipd3N4_-infected rhesus macaques after 5 years of infection [45]. Subsequently, we successfully isolated the J038 lineage of V2-specific bnAbs from donor G1015R using a pair of V2-specific wild-type/mutant differential hooks [46,47]. However, J038 did not neutralize some HIV-1 strains (e.g., X1632) because it relied heavily on the N156 and N160 glycans. Thus, the neutralization breadth of the G1015R plasma was not fully recapitulated by the J038 lineage bnAbs. Our previous epitope mapping experiments showed that neutralization activities were also against other HIV-1 epitopes [47]. Over time, the virus accumulated mutations not only in the V1V2 region, but also in the V3 loop, CD4bs, and gp120-gp41 interface regions, indicating that these regions were under selection pressure by nAbs in G1015R [47].

To isolate and characterize bnAbs with other specificities, we synthesized the protein hook based on the stable BG505 uncleaved prefusion-optimized (UFO) trimer [48]. We obtained J038-like V2-specific antibodies JT15 and JT16, as well as a new nAb JT18, with the V3-specificity. Although the neutralization breadth of JT18 was narrower than the J038 lineage bnAbs, the combination of JT18 and J038 antibodies greatly increased the neutralization breadth and potency, fully recapitulating the neutralization breadth of the plasma in G1015R. These findings may have important implications in the design of new HIV-1 vaccines that can induce multiple nAbs with different specificities to increase the overall neutralization breadth.

## 2. Results

### 2.1. Isolation of HIV-1 Specific mAbs using a Stable UFO Env Trimer from a SHIV-Infected Macaque

**Figure 1:**
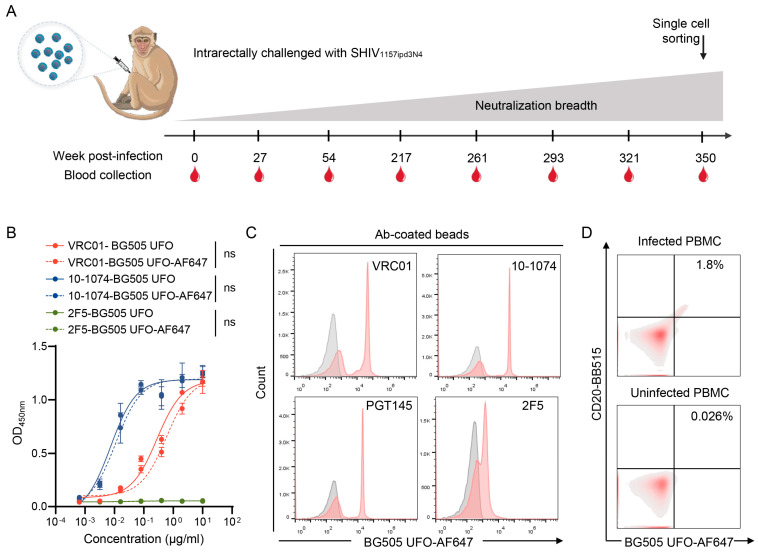
Validation of the new stable trimer hook. (**A**) Schematic diagram of the blood collection of the SHIV_1157ipd3N4_-infected G1015R. PBMCs from week 350 were used to obtain the VH and VL fragments. (**B**) Determination of binding affinity of the new BG505 UFO Env hook by ELISA. VRC01 (CD4bs), 10-1074 (V3 glycan), 2F5 (MPER) were used to determine the binding affinity. Each antibody was assayed in triplicates. ns, not significant (two-way ANOVA). (**C**) The binding specificity of the BG505 UFO-AF647 hook was determined with beads coated with 2F5, PGT145, 10-1074, or VRC01 by flow cytometry. The unconjugated negative beads are shown in gray and the experimental group incubated with the BG505 UFO-AF647 hook and the beads conjugated to different antibodies are shown in red. (**D**) Evaluation of the specific binding of the BG505 UFO-AF647 hook using uninfected and infected PBMCs.

A number of studies have shown that polyclonal antibodies targeting different epitopes exist in most HIV-1-infected individuals, but it is unclear if such mAbs can also be elicited in NHPs [21,25,27]. After the macaque G1015R was infected with SHIV_1157ipd3N4_ for 5 years, broad neutralization activity was detected in its plasma (Figure 1A) [45]. Soluble trimers have been widely used as sorting hooks to isolate bnAbs, without being biased toward any particular specificities, from HIV-1-infected individuals [49,50,51]. Thus, to better understand the repertoire of bnAbs in G1015R, we generated a stable BG505 Env UFO trimer hook as previously described to isolate other neutralizing antibodies [49]. Since this stable UFO trimer configuration preserves Env secondary conformation well, it will more likely allow us to pick up novel mAbs that depend on the conformation epitopes on the native Env trimer [48]. The BG505 env UFO gene was modified by adding an Avi tag at the C terminus and the BG505 Env UFO-Avi tag trimer protein was purified by lectin affinity chromatography and size exclusion chromatography (SEC) (Figure S1A). The BirA ligase and the streptavidin-AF647 were added to label the BG505 UFO trimer. The final labeled protein was validated by Native-PAGE. To understand if different labelling methods will affect the tertiary structure of the BG505 UFO hook proteins, we made three different BG505 UFO hook proteins labeled with biotin alone, biotin–streptomycin, or biotin–streptomycin–fluorescent, respectively. Blue native PAGE analysis showed that all different modifications did not affect the trimer conformation of the BG505 UFO hook proteins (Figure S1B).

To carefully characterize the newly generated BG505 Env UFO trimer hook, we performed a number of assays to validate its quality and specificity. We first determined the binding ability of the trimer protein to three well-characterized bnAbs VRC01 (CD4bs), 10-1074 (V3), and 2F5 (MPER), with or without fluorescence labelling by ELISA. The fluorescence labeling did not affect the binding of BG505 UFO trimer to all three antibodies (Figure 1B). Next, we used antibody-coated beads to simulate cells with B cell receptor (BCR) on their surface. The test bead population consisted of half of the unlabeled beads and half of the beads labelled with one of the mAbs (VRC01, 10-1074, PGT145, and 2F5). The analysis showed that the fluorescence labeled BG505 UFO trimer bound to about half of the bead population coated with VRC01, 10-1074, or PGT145 (Figure 1C). 2F5, which does not bind to the BG505 UFO trimer, did not show specific binding to the antibody-coated beads. Finally, we used the new hook to analyze infected and uninfected peripheral blood mononuclear cells (PBMCs) and found that it bound infected cells nearly 70 times (1.8%/0.026%) better than uninfected cells (Figure 1D). The infected PBMC samples were obtained from the SHIV_1157ipd3N4_-infected rhesus macaque G1015R [45]. These results demonstrated that the stable BG505 UFO trimer hook is specific, stable, and easy to use.

**Figure 2:**
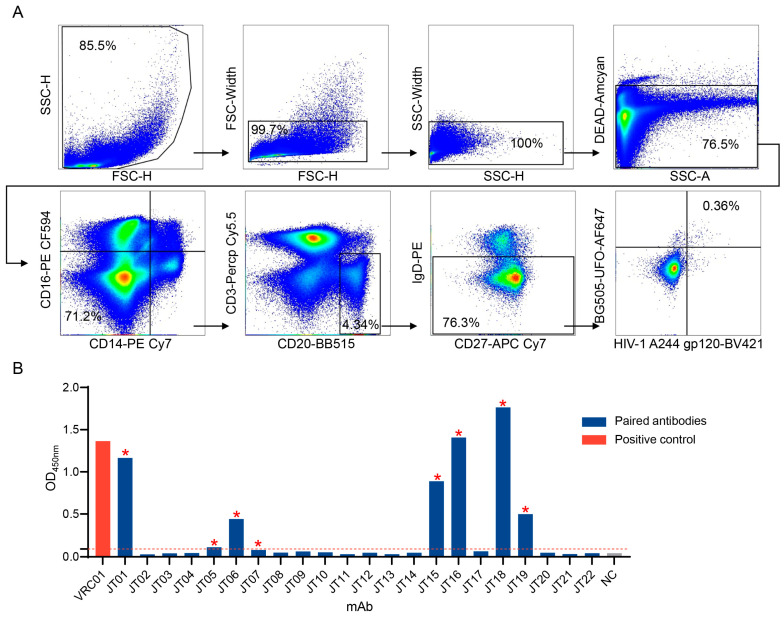
Single cell sorting strategy and identification of HIV-1 Env specific binding antibodies. (**A**) Single cell sorting by fluorescence-activated cell sorting (FACS). Individual HIV-1 Env specific memory B cells from G1015R were identified for their positive staining with BG505 UFO-AF647 and HIV-1 A244 gp120-BV421 hooks. PBMCs were analyzed using the samples collected by 350 weeks after G1015R was infected with SHIV_1157ipd3N4_. (**B**) Determination of specific binding of antibodies to HIV-1 Env. The newly characterized antibody genes were expressed and their binding to autologous SHIV_1157ipd3N4_ gp120 was determined by ELISA. Asterisks indicate the gene families from which their members specifically bound to Env.

We next used the new BG505 UFO trimer hook and a gp120 monomer hook to sort HIV-1 Env specific memory B cells from the PBMCs in G1015R (Figure 2A). The use of both new BG505 UFO trimer hook and our previous gp120 monomer could further increase the chance to identify HIV-1 Env specific antibodies. The trimer-specific memory B cells accounted for 0.055‰ of the total PBMC population. The single sorted positive cells were individually lysed, and the mRNA was reverse transcribed, then the variable region (V) of both heavy and light chains (VH and VL) of the antibody was amplified by single-cell PCR [46]. We obtained 22 antibody V genes with paired VH and VL chains (Tables S1 and S2). They were expressed as recombinant IgG by adding the constant region to the VH and the CMV promoter to both VH and VL fragments. The expressed antibodies were tested for their binding ability to autologous gp120 monomer protein. Six antibodies (JT01, JT06, JT15, JT16, JT18, and JT19) were found to strongly bind specifically to gp120 and two antibodies (JT05 and JT07) only weakly bound to gp120 (Figure 2B). Sequence analysis showed that they were from six different gene families. The pairs of JT05/JT06 and JT15/JT16 were each derived from a distinct gene family, while all other four antibodies were each derived from different gene families (Table 1). The average length of the heavy chain CDR3 was 15.2 aa (12–18) and the average of somatic hypermutation (SHM) was 14.3% (11-20.49) (Table 1). Interestingly, JT15 and JT16, which shared the same lineage with J038, had the longest CDR3 length (18 aa) and the highest SHM rate (18.06% and 20.49%, respectively) [46].

### 2.2. Comparison of Antibody Sequences Obtained Using Different Env Hooks

**Figure 3:**
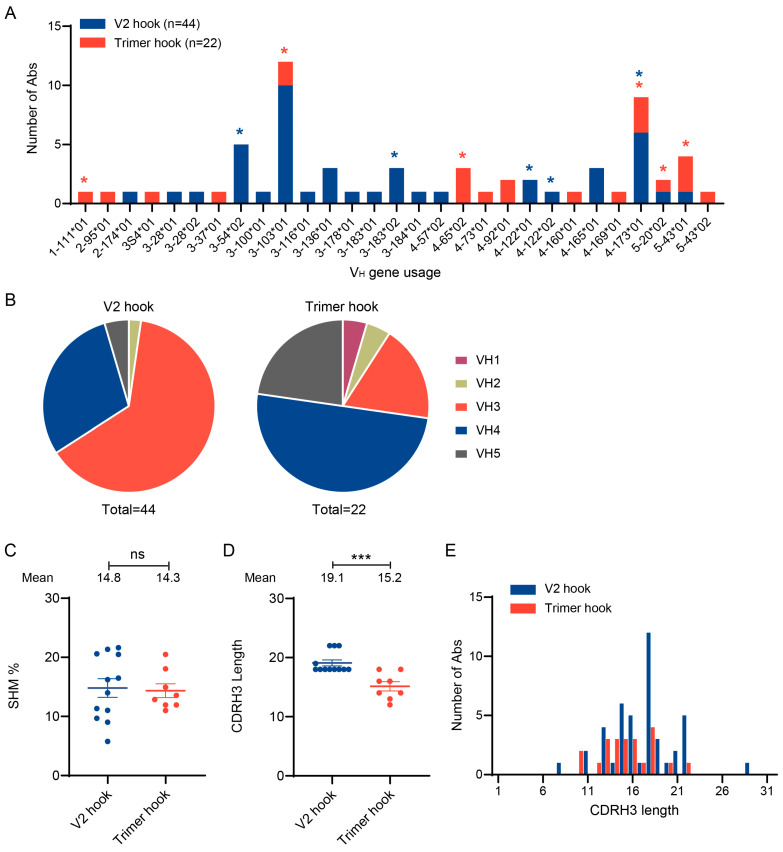
Antibodies from disparate gene families were isolated with different hooks. (**A**) Comparison of the gene families of antibody heavy chain variable regions isolated by two different hooks (V2 and UFO trimer). Asterisks indicate the gene families from which members can specifically bind to Env. (**B**) Comparison of the proportions of the gene families isolated by V2 and trimer hooks. (**C**) The differences in SHM of the antibodies isolated by two different hooks. ns, not significant (Student’s *t*-test). (**D**) Differences in HCDR3 lengths of the antibodies isolated by two different hooks and HCDR3 length. ***, *p* < 0.001 (Student’s *t*-test). (**E**) HCDR3 lengths of antibodies isolated from G1015R at week 350.

We obtained many antibody sequences using the V2 differential hook in our previous study [46] and a mixture of the UFO trimer hook and gp120 monomer hook in this study (Tables S1 and S2). To understand if different hooks select antibodies from different gene families, we compared all the VH sequences from both studies: 44 from the V2 deferential hook and 22 from the UFO timer hook. Most of the gene members identified with the V2 and trimer hooks were unique (Figure 3A). Among the 29 gene members detected by both hooks, only four members (VH3-103, VH4-173, VH5-20, and VH5-43) were detected by both the V2 and trimer hooks. Overall, VH3 were predominant (64%) among all the family members detected by the V2 hook, while VH4 accounted half (50%) of the family members detected by the trimer hook (Figure 3B). The SHM rates were similar among all the binding antibodies obtained by the UFO trimer and V2 differential hooks (Figure 3C). However, the average of the HCDR3 length was significantly longer among the binding antibodies obtained by the V2 differential hook (19.1 aa) than those obtained by the UFO trimer hook (15.2 aa) (Figure 3D). Previous studies have shown that many bnAbs, such as those with V2-apex specificity, rely on a long HCDR3 region for high affinity binding to Env [10,52,53]. Thus, the difference in the HCDR3 length may indicate that antibodies obtained with different hooks are derived from different antibody repertoires with different HIV-1 binding specificities. The average length of HCDR3 in the initial B cell repertoire is only 14.81 aa in monkeys, which is much shorter than that in humans (16.01 aa) [54,55]. The average HCDR3 length of all antibodies isolated from G1015R was 16.58 aa (Figure 3E), indicating that the antibodies might have undergone maturation during the 350 weeks of infection. Thus, these results suggest that the UFO trimer hook has identified some new nAbs that are different from those obtained with the V2 differential hook.

### 2.3. Broad Neutralization Properties of Newly Obtained mAbs

**Figure 4:**
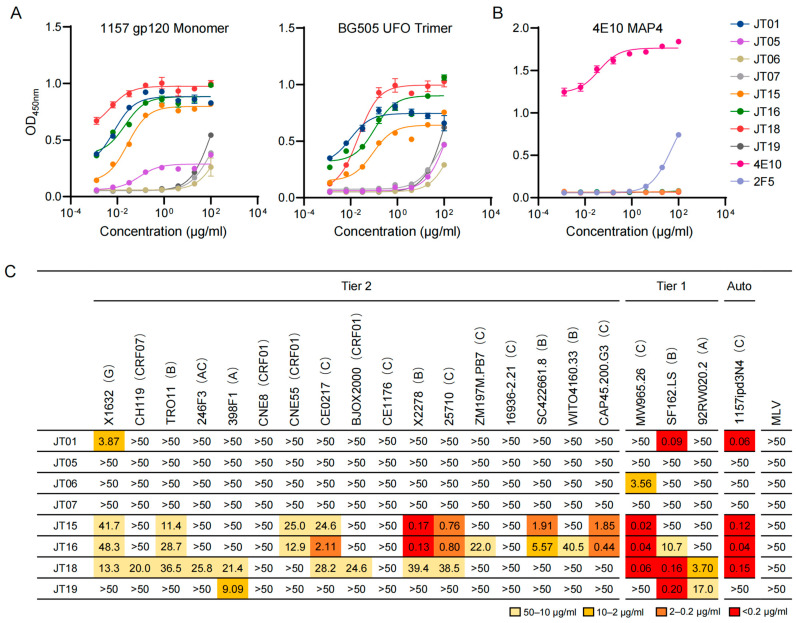
Functional analyses of newly isolated antibodies. (**A**) Determination of binding ability of the newly isolated antibodies to SHIV_1157ipd3N4_ gp120 monomer and BG505 UFO trimer. (**B**) Binding to 4E10 MAP4 peptide by four antibodies JT01, JT15, JT16, and JT18. (**C**) Neutralization IC_50_ of newly isolated antibodies. All eight mAbs were assayed against with 17 tier 2 viruses, 3 tier 1 viruses, the autologous SHIV_1157ipd3N4_ virus, and murine leukemia virus (MLV). The different colors represent the neutralization strength.

To more accurately determine the binding ability of the eight-new antibodies, we expressed them as previously described [46] and tested them with the autologous gp120 monomer and the heterologous BG505 UFO trimer. Four antibodies (JT01, JT15, JT16, JT18) showed high binding ability to both the autologous gp120 monomer and the heterologous BG505 UFO trimer (Figure 4A). JT05 only weakly bound to the autologous gp120 monomer protein. All three other antibodies (JT06, JT07, JT19) only weakly bound to both antigens. Four mAbs (JT01, JT15, JT16, JT18) had higher EC_50_ (<1 μg/mL) to both Env proteins (Table 2). The BG505 UFO protein contains a partial residue 661 to 664 of the MPER epitope [48]. However, none of these four antibodies bound to the MPER epitope (Figure 4B), indicating that their recognition sites are located outside of the MPER region. We next determined their neutralizing activity against seventeen tier 2 viruses, three tier 1 viruses, and the autologous SHIV_1157ipd3N4_ virus. Four mAbs (JT01, JT15, JT16, JT18) that had high binding ability potently neutralized the autologous SHIV_1157ipd3N4_ and some or all neutralized tier 1 viruses. JT15 and JT16 had similar neutralization profiles, neutralizing eight (47.1%) and ten (58.8%) tier 2 viruses, respectively (Figure 4C). Although JT18 had a similar neutralization breadth of 52.9% (9/17), it neutralized a set of viruses distinct from those neutralized by JT15 and JT16. JT01 only neutralized one tier 2 virus (Figure 4C).

**Figure 5:**
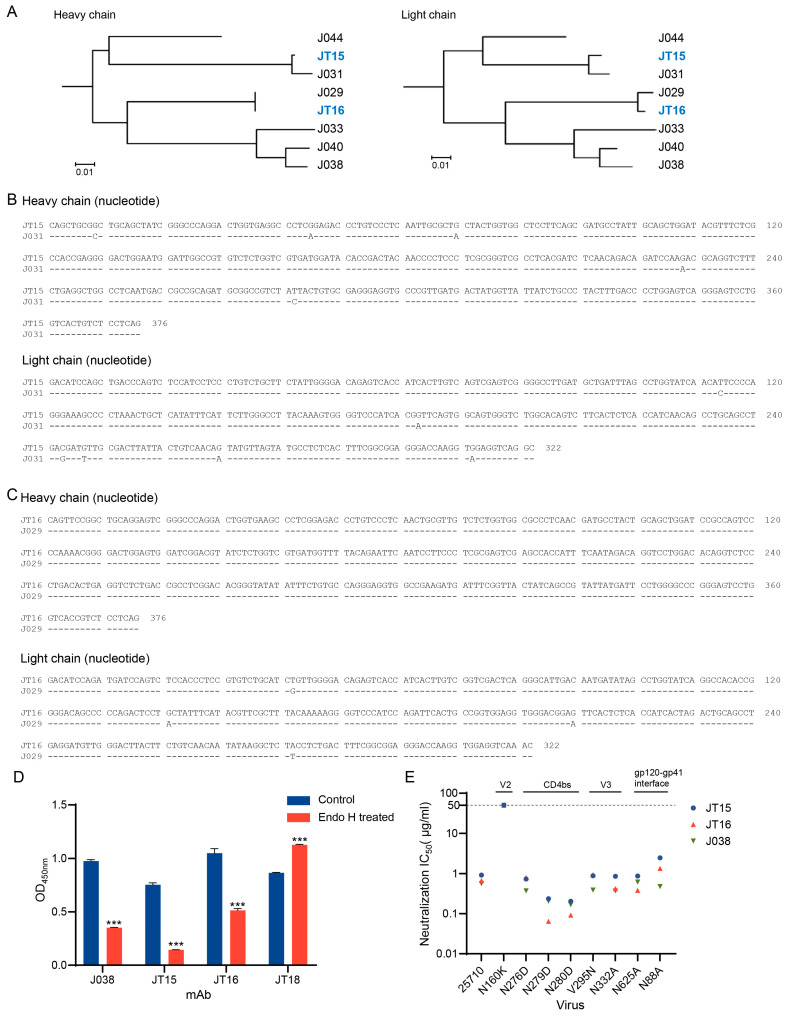
Functional analyses of JT15 and JT16 antibodies. (**A**) Phylogenetic tree analysis of the nucleotide sequences of the J038 lineage bnAbs. The newly obtained antibodies JT15 and JT16 are highlighted in blue. Alignment of antibody sequences from different hooks. (**B**) Alignment of heavy- and light-chain sequences between JT15 and J031 as amino sequences. (**C**) Alignment of heavy- and light-chain sequences between JT16 and J029 as amino acid sequences. (**D**) The impact of deglycosylation on binding to antibodies. The glycans on the BG505 UFO trimer were removed by Endo H enzyme. The comparison between treated and untreated groups was analyzed using a paired *t*-test. ***, *p* < 0.001. (**E**) Determination of potential neutralization targets. Neutralization susceptibility of wild type 25710 and its mutants were analyzed against JT15, JT16, and J038.

Phylogenetic analysis confirmed that JT15 and JT16 belong to the same V2-specific J038 bnAb lineage that we previously obtained from the same macaque G1015R. JT15 and JT16 were also highly similar to J031 and J029, respectively, which are members of the J038 lineage (Figure 5A). There were only a few nucleotide differences in both the VH and VL chains between JT15 and J031 or J029 and JT16 (Figure 5B,C). JT15 and J031 had similar neutralization profiles, suggesting that the different amino acids had minimal impact on the antibody’s neutralization efficacy (Figure S2). To investigate if the binding of these mAbs is dependent on glycans on the Env surface like J038, we treated the BG505 UFO trimer with deglycosylase Endo H and measured the binding ability of these antibodies before and after the treatment. The binding of JT15 and JT16 to the trimer significantly decreased after treatment, similar as J038 that heavily depends on high-mannose for its binding (Figure 5D). The binding of JT18 was increased after the treatment, indicating that its target epitopes are different from those of J038. To determine the potential targets of JT15 and JT16, we analyzed their neutralization ability with a set of mutants from a tier 2 virus 25710 which was potently neutralized by both mAbs (Figure 5E). Both JT15 and JT16, like J038, completely lost their ability to neutralize the N160K mutant even at the highest tested concentration (50 µg/mL). This indicates that JT15 and JT16 antibodies recognize a similar epitope as the previously identified J038 lineage antibodies [46].

### 2.4. JT18 Recognizes a Distinct V3 Epitope

**Figure 6:**
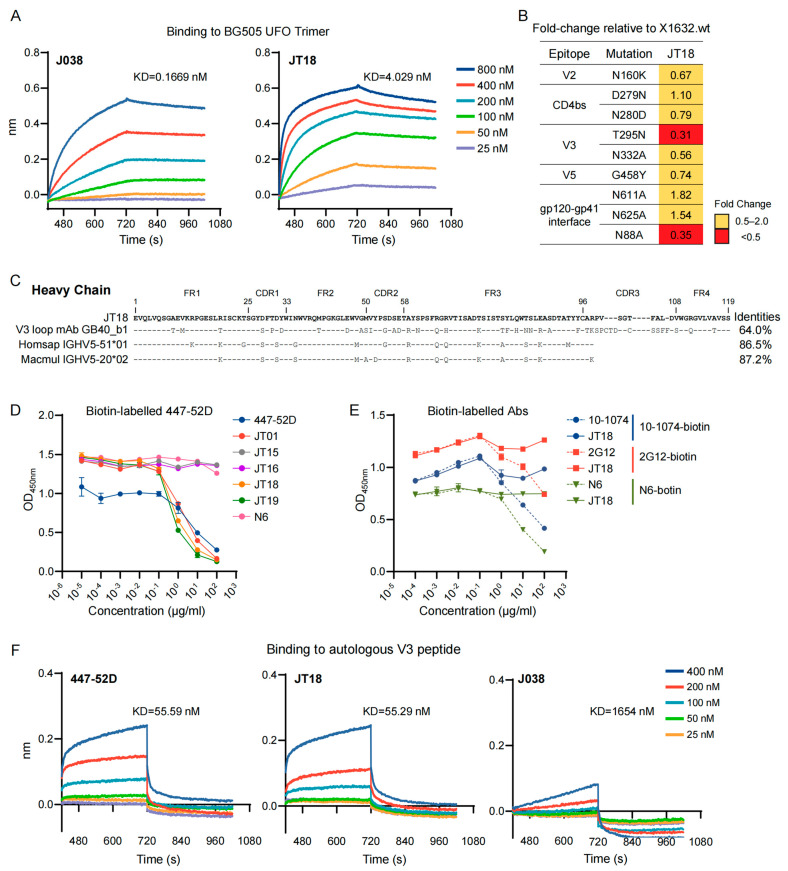
JT18 recognized an epitope in the V3 loop region. (**A)** J038 and JT18 had a strong binding ability to BG505 UFO trimer. Dynamic binding and dissociation of antibodies (20 μg/mL) to serially diluted BG505 UFO trimer was determined by biolayer interferometry (BLI). (**B**) Determination of potential neutralization targets of JT18. Neutralization susceptibility of wild type X1632 and its mutants were analyzed against JT18. (**C**) Alignment of the antibody heavy chain amino acid sequences. Sequences from mAb GB40_b1 (targeting V3 loop), Homsap IGHV5-51*1 F, and Macmul IGHV5-20*2 F were compared to JT18. (**D**) Competition of newly characterized antibodies with 447-52D. Binding of biotin-labelled 447-52D was competed by five new antibodies JT01, JT15, JT16, JT18, and JT19. Each assay was performed in triplicates. (**E**) Competition binding between JT18 and biotin-labeled antibodies. Binding of JT18 to Env was competed with biotin-labeled 10-1074, 2G12, and N6. Experiment was repeated twice and results from one repeat is shown. (**F**) Binding affinity of JT18 to the autologous V3 peptide. Binding affinity of 447-52D, JT18, and J038 to a V3 peptide was determined by BLI analysis.

The biolayer interferometry (BLI) results showed that the binding affinities of antibodies were at 0.167 nM for J038 and 4.029 nM for JT18 (Figure 6A). JT18’s binding affinity to the heterologous BG505 UFO trimer was weaker than J038. To determine the potential targets of JT18, we determined their neutralization ability with a set of mutants from a tier 2 virus X1632. Compared to the X1632.wt, none of the mutants significantly reduced the neutralization titer by JT18 (Figure 6B). This suggests that JT18 might recognize and neutralize HIV-1 through an epitope that is not present among those mutants. Sequence analysis showed that JT18 had a high similarity (86.5%) with human IGHV5-51*1 (Figure 6C). Other studies indicated that the human IGHV5-51*1 is the gene family for several human V3-specific broadly neutralizing antibodies [38,56]. GB40-b1, which is a mAb isolated from a Chinese rhesus macaque infected with SHIV_SF162_, targets the V3 loop and has a high similarity with IGHV5-51*1, and competitively blocked the binding of 447-52D to Env [38]. These results suggest that JT18 might recognize a similar epitope in the V3 loop region.

To determine if the newly characterized mAbs bound to V3, we tested five antibodies (JT01, JT15, JT16, JT18, JT19) that showed neutralization activity against at least one tier 2 virus using the competitive binding analysis with the biotin-labelled 447-52D antibody. The addition of the 447-52D antibody significantly competed the binding of 447-52D to BG505 UFO trimer, whereas the addition of N6 antibody did not affect the binding of 447-52D (Figure 6D). JT01, JT18, and JT19 could block the biotin-labelled 447-52D binding to Env, even more effectively than unlabeled 447-52D itself (Figure 6D). JT15 and JT16, which recognize the V2 epitope, did not affect the binding of the biotin-labelled 447-52D to Env as the control antibody N6 that binds to CD4bs. To investigate if JT18 still bound to other possible sites, we did the competitive blocking experiments against V3-glycan-dependent antibody 10-1074, glycan-dependent antibody 2G12, and CD4bs-specific antibody N6. JT18 did not affect the binding of all three antibodies (Figure 6E). These results indicate that JT18 neutralized HIV-1 through binding to an epitope in V3, but not dependent on the glycan in V3 like 10-1074. We next determined if JT18 could specifically bind to the V3 peptide. According to the research results against the database [57,58], a V3 peptide (^305^KSISIGPGQAI^317^) from the autologous SHIV_1157ipd3N4_ Env sequence was synthesized to measure the specific binding of JT18. JT18 effectively bound to this V3 peptide at a nanomolar level, similar to 447-52D (Figure 6F). In contrast, J038 showed little binding to the V3 peptide (Figure 6F). Taken together, JT18 represents a distinct nAb that broadly neutralizes tier 2 viruses through an epitope in V3.

### 2.5. A JT18 Escape Mutation Is Compensated by a Separated Mutation in V3

**Figure 7:**
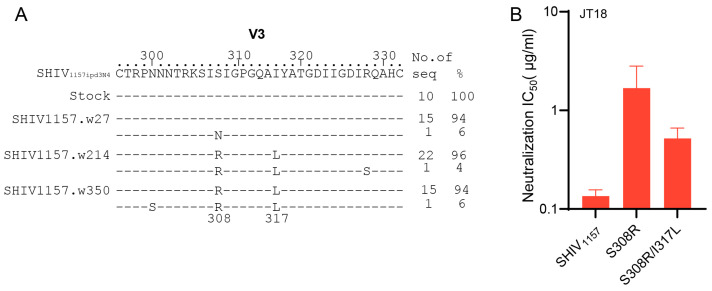
Escape and compensatory mutations to JT18. (**A**) Alignment of the V3 amino acid sequences. The V3 sequences from different time points were compared to the sequence from the inoculation virus stock SHIV_1157ipd3N4_. (**B**) Identification of neutralization escape mutation. Neutralization susceptibility of the wild type SHIV_1157ipd3N4_ and its mutants were analyzed against JT18. Experiment was repeated twice.

We did not detect neutralization activity against V3 in G1015R using two glycan associated mutants V295N and N332A in our previous study [47]. We also did not identify any mutations at both sites in V3 among all viral sequences throughout the 350 weeks of infection of G1015R, confirming that there was no immune selection pressure on these sites. However, isolation of V3-specific mAb JT18 demonstrated that a broad nAb was indeed induced in G1015R. Examination of the V3 sequences showed two mutations S308R and I317L within the tested V3 peptide sequence. Both mutations were not detected in viral sequences by week 27 but fixed in the viral population at weeks 214 and 350 (Figure 7A). To investigate if these mutations were selected by JT18, we introduced both mutations individually or together into the autologous SHIV_1157_ *env* gene. Pseudoviruses were successfully obtained for the S308R mutant and the S308R/I317L double mutant, but the I317L mutant failed to produce infectious viruses after repeated attempts. The S308R mutant was significantly more resistant to JT18 than the SHIV_1157_.wt, requiring 13 times more JT18 to be neutralized (Figure 7B). Interestingly, the double mutant S308R/I317L was more sensitive to neutralization than the mutant S308R, indicating that the additional mutation I317L restored neutralization susceptibility of the escape S308R mutant.

### 2.6. Recapitulation of Plasma Neutralization Breadth by Both JT18 and J038

**Figure 8:**
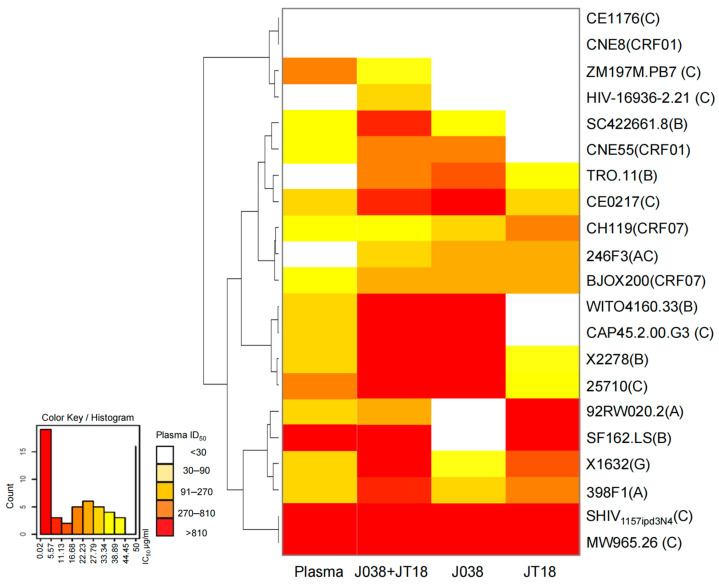
Recapitulation neutralization breadth of the serum by the combination of JT18 and J038. Heat map analysis of neutralization of 21 viruses by JT18, J038, J038, and JT18 mix, and plasma from G1015R.

JT18 as a V3-specific antibody was able to neutralize two tier 1 viruses SF162 and 92RW020, which were resistant to the V2-specific J038 lineage antibodies [46] (Figure 4C). This suggests that both lineage antibodies might complement each other to recapitulate the neutralization breadth in the plasma in G1015R. Thus, we mixed J038 and JT18 antibodies at equal amount and compared its neutralization activity with those of individual antibodies and the plasma. When tested against all 21 viruses, J038 and JT18 each failed to neutralize some viruses: SF162 and 92RW020 for J038; and SC422, CNE55, WITO, and CAP45 for JT18 (Figure 8 and Figure S3). However, the J038 and JT18 mixture could neutralize all six viruses. Interestingly, while ZM197 or 16936 was not neutralized by J038 or JT18 each alone, both viruses could be neutralized by the combination of both J038 and JT18, suggesting that the two antibodies could synergize to neutralize the viruses that were not neutralized by each antibody alone (8 and S3), similar to the synergized effect between antibodies targeting two different epitopes [27,59,60,61,62,63]. Since both lineage bnAbs were present at the same time, after bnAbs from one lineage bound and neutralized a virus, this could alter the Env conformation and rendered the virus more susceptive to neutralization by the bnAbs from a different lineage. As a result, additional viruses were neutralized than the combined number of viruses neutralized by bnAbs from both lineages. Overall, JT18 neutralized 61.9% (13/21) of the viruses and J038 neutralized a little more at 71.4% (15/21), while the combination of both could neutralized 90.5% (19/21) of the viruses. This is even more than the neutralized viruses (76.2%) by the plasma from the macaque G1015R (8 and S3). Taken together, the combination of both JT18 and J038 recapitulated the neutralization breadth of their cognate plasma, suggesting that they were likely the major neutralizing lineage bnAbs in G1015R.

## 3. Discussion

To better understand what nAbs lineages are present in an infected host and if they can recapitulate the neutralization activities will have important implications in HIV-1 vaccine design. Multiple nAb lineages can be identified in the same infected individuals [21,22,23,24,25,26,27]. Here, we isolated two different lineage bnAbs from one SHIV-infected Chinese rhesus macaque using the BG505 UFO Env trimer hook and gp120 monomer hook: JT15 and JT16 from the same J038 lineage, as we previously identified, and JT18 from a distinct lineage. The former targeted the V2-apex and the later bound to a V3 epitope in Env. Each lineage bnAbs neutralized a distinct set of tested viruses, but both together fully recapitulated the neutralization breadth of the cognate plasma.

We used the BG505 UFO Env trimer hook to identify two bnAbs, JT15 and JT16, which belong to the J038 bnAb lineage that target V2-Apex of Env. Identification of the bnAbs from the same lineage using the combination of the newly developed BG505 UFO Env trimer and gp120 monomer hooks on a separated occasion indicates that this bnAb lineage is predominant in the infected host. This lineage of bnAbs has unique features that make it an ideal lineage to induce by HIV-1 Env vaccines: neutralization of autologous SHIV_1157_ by its unmutated common ancestor, a better contact with Env trimer through the up V2 position, the short HCDR3 (17 aa), low somatic hypermutation rate (15%), and the predominance in the host. These indicate that they naturally occur at a high frequency and can be relatively easy to induce by vaccines.

JT18 is a V3-specific bnAb. Although it was not as broad or potent as the J038 lineage bnAbs, the identification of two bnAb lineages from the same host demonstrate that different hook designs may favor bnAbs with different specificities, and the use of multiple different hooks will likely help to obtain bnAbs with distinct specificities. Identification of Abs that were clonally related to previously isolated bnAbs from the same animal using a targeted differential hooks demonstrates the feasibility to use the BG505 UFO trimer as an immunogen to elicit broad neutralization activity.

We previously screened the V3-specific bnAbs using a V3 mutant virus with mutations at glycosylation sites at both ends of the V3 loop, but did not identify such nAbs in G1015R [47]. Sequence analysis showed no mutations at both sites throughout the 350 weeks of infection, strongly suggesting that bnAbs that target V3 glycans were not elicited in this macaque. However, JT18, a V3-specific bnAb that targets the V3 crown, was successfully isolated using the BG505 UFO Env trimer hook. JT18 selected a strong escape mutant S308R. It is possible that I317L has such adverse effects on envelope folding that it affects the overall neutralization properties of the envelope and does not specifically ‘restore’ partial sensitivity to JT18. The dual mutation mutant S308R/I317L can be equally neutralized like the wild type virus, suggesting that it can be more efficiently bound by the JT18 and therefore might play a critical role in driving the maturation of the JT18 lineage bnAbs as we found in the cooperation between the CH235 and CH103 lineage bnAbs [64].

Neutralizing Abs targeting V3 are relatively easy to induce [19,65,66,67,68]. The V2-specific bnAbs like the ones from the unique J038 lineage described above can also be more easily induced. Importantly, both J038 and JT18 together can complement each other and fully recapitulate the neutralization breadth in their cognate plasma. One limitation in our study is the relatively small number of viruses used to determine neutralization breadth. If both lineages of bnAbs can be elicited by a vaccine, it would be more likely to successfully prevent HIV-1 infection. Further studies in this area may open an avenue to develop a new vaccine approach for control of continuous spreading of HIV-1 infection.

## 4. Materials and Methods

### 4.1. Construction and Characterization of an HIV-1 Env BG505 UFO Hook

BG505 UFO was optimized as a stable trimeric structure [48]. To use it as sorting hook, an Avi tag (GLNDIFEAQKIEWHE) was added at its C terminus as previously described [49,69]. 293-6E cells (ATCC, Manassas, VA, USA) were transfected with the BG505 UFO-Avi tag plasmid. At 72 h after transfection, the cell culture supernatants were harvested and purified through lectin affinity chromatography and size exclusion chromatography (SEC). The trimer proteins were biotinylated using BirA ligase (Avidity Biosciences, San Diego, CA, USA) and then conjugated with streptavidin-AF647 (Bioss, Beijing, China), as described previously [70]. The final hook was assessed by binding to various well characterized Abs, as described below. BG505 UFO-streptavidin-AF647 or BG505 UFO was used to coat the wells in the ELISA plate at 4 °C overnight. After the wash, each of the serially diluted (at 1:5 ratio) antibodies VRC01, 10-1074, and 2F5 was added to the well. The specific binding of each antibody to each protein was determined on a ELX800 Microplate reader (Bio Tek, Winooski, VT, USA). Validation of specific binding of the generate BG505 UFO-streptavidin-AF647 hook by flow cytometry using antibody-coupled compensation beads. Briefly, the UltraComp eBeads™ Plus Compensation Beads (Thermo Fisher, Waltham, MA, USA) were incubated with 2 μg/mL of VRC01, 10-1074, PGT145, or 2F5. After washing twice with PBS, BG505 UFO-streptavidin-AF647 was then incubated with the beads and analyzed on BD Accuri™ C6 Plus Flow Cytometer (BD Biosciences, Franklin Lakes, NJ, USA).

The uninfected human PBMC samples were obtained from the Jilin University School Hospital, and the PBMC samples collected at week 350 post-infection (viral load at 6.6 × 10^5^ copies/mL) were obtained from rhesus macaque G1015R, which was infected with SHIV_1157ipd3N4_, as described in our previous report [45]. To analyze the specificity of the BG505 UFO-AF647 hook, multiple fluorescent antibodies to IgD-PE (Southern Biotech, Clone IA6-2), CD3-PE Cy5 (BD Biosciences, Clone UCHT1), CD20-BB515 (BD Biosciences, Clone 2H7), and BG505 UFO-AF647 were incubated with the samples and analyzed by flow cytometer. All human samples were collected with informed consent under clinical protocols approved by the Jilin University School Hospital institutional review board (IRB).

### 4.2. Single B-Cell Sorting

Peripheral blood lymphocytes were stored at −80 °C, originally from a Chinese rhesus macaque G1015R which was infected with SHIV_1157ipd3N4_ for 350 weeks [45,46,47]. Frozen PBMCs were thawed in RPMI 1640 medium containing 10% FBS and then washed and re-suspended with 5% FBS-PBS. A multicolor fluorescent antibody mixture containing DEAD-Amcyan, CD16-PE CF594 (BD Biosciences, Clone 3G8), CD14-PE Cy7 (BD Biosciences, Clone M5E2), CD3-Percp Cy5.5, CD20-BB515, IgD-PE, CD27-APC Cy7 (BD Biosciences, Clone M-T271), BG505 UFO-AF647, and HIV-1 A244 gp120-BV421 was prepared and incubated with the cell suspension at room temperature in the dark for 45 min. After washing and re-suspending the cells with 5% FBS-PBS, the live cell population in the stained cell suspension was used to directly sort single cells into individual wells in a 96-well PCR plate containing reverse transcription lysis buffer.

### 4.3. Single B-Cell RT-PCR

The variable genes of heavy and light chains were amplified as previously described [46,71]. Briefly, the cDNA was made by adding 150 ng/µL Random Hexamers (Qiagen, Germantown, MD, USA), 4 μM High Pure dNTPs (TransGen Biotech, Beijing, China), and 200 U Superscript III (Invitrogen, Grand Island, NY, USA) to the RT lysis buffer. Then, the obtained cDNA template was amplified by two rounds of nested PCR using the complete set of primers to amplify the variable regions of the rhesus macaque antibodies [72]. To minimize the error rate during PCR, High Fidelity Taq DNA polymerase (Invitrogen) and KOD Polymerase (TOYOBO, Osaka, Japan) were used for amplification. The amplified IgG heavy- and light-chain variable region sequences were analyzed using the international ImMunoGeneTics information system (IMGT) V-quest webserver (https://www.IMGT.org, accessed on 8 March 2023). 

### 4.4. Expression and Purification of Proteins

The CMV promoter, the amplified antibody variable regions, and the constant regions were assembled together by overlap PCR [72]. Subsequently, the heavy- and light-chain linear expression fragments were co-transfected into HEK293T cells from ATCC (Manassas, VA, USA) for small-scale expression. After 72 h, the supernatant was collected to determine the specificity of the antibodies by their binding to autologous SHIV_1157ipd3N4_ gp120. The genes that expressed antibodies binding to autologous SHIV_1157ipd3N4_ gp120 were cloned into the pcDNA3.1^+^ vector for subsequent protein expression.

Antibody proteins were produced by co-transfecting the full heavy- and light-chain plasmids into Expi293Fcells (Thermo Fisher). In brief, Expi293F cells were passaged for more than three generations, ensuring >90% cell viability. Cells (7.5 × 10^7^) were transferred to 25.5 mL of Expi293^TM^ Expression Medium (Thermo Fisher). The heavy- and light-chain plasmids (30 µg) were mixed at a 1:2 ratio and then added to 1.5 mL of Opti-MEM (Thermo Fisher) while 81 μL of transfection reagent was added to 1.5 mL of Opti-MEM. Both mixtures were incubated at room temperature for 5 min. After the transfection reagent mixture was added to the plasmid mixture, the combined mixtures were gently mixed and incubated at room temperature for 20 min. The transfection complex was slowly dropped into the cell culture supernatants. After incubation for 20 h, 150 μL of Enhancer 1 and 1.5 mL of Enhancer 2 were added. When the cell viability was < 60%, the culture supernatant was harvested.

The harvested cell supernatant was centrifuged at 3500 rpm at 4 °C and for 30 min to remove cell debris and then filtered through a 0.22 μm membrane. After equilibration of the protein A agarose (Thermo Pierce, Waltham, MA, USA) with the binding buffer, the harvested supernatant and protein A agarose were mixed together and incubated at 4 °C in a shaker overnight. The mixture was loaded onto the purification column. After washing, the column was eluted with the elution buffer. The pH of the eluates was adjusted to neutral by adding 100 μL of neutralization buffer per 1 mL of the eluate. Finally, the antibody proteins were concentrated with a 30 kD ultrafiltration tube and stored at −80 °C.

### 4.5. Enzyme-Linked Immunosorbent Assay

The autologous gp120 monomer, BG505 trimer or 4E10 MAP4 [73] was used to coat plates (200 ng per well) at 4 °C overnight. After blocked with 200 µL PBS with 3% BSA for 2 h at 37 °C, 1:5 serially diluted antibodies (100 µL) were added and incubated at 37 °C for 1 h. The horseradish peroxidase labeled rabbit anti-monkey IgG antibody (Bioss) was added and incubated at 37 °C for 45 min. Plates were washed three times with PBST (0.1% Tween 20 in PBS) and then 100 µL of tetramethylbenzidine (TMB) substrate was added to each well. Lastly, the reaction was stopped by adding 50 µL of 2 M sulfuric acid. The absorbance values were determined at a wavelength of 450 nm on a ELX800 Microplate reader.

The glycan-dependent binding was determined as below. A mixture of 20 µg of protein, 2 µL of Glyco Buffer 3, and 5 µL Endo H (New England Biolabs, Ipswich, MA, USA) in 20 µL was incubated at 37 °C for overnight. The treated protein was then used to coat the polystyrene plates. After the plates were blocked using PBS with 3% BSA, the antibodies at concentrations of 10 µg/mL or 100 µg/mL were added. Subsequently, HRP-conjugated secondary antibodies and TMB substrate were added sequentially and incubated for a period of time. After 2 M H_2_SO_4_ was added, the reaction was stopped. The effect of deglycosylation was determined by measuring absorbance values.

For the competitive ELISA assay, 200 ng of the BG505 UFO trimer protein was used to coat each well in the plate and incubated at 4 °C overnight. After blocking with 200 μL PBS containing 3% BSA at 37 °C for 2 h, the competitive antibody, serially diluted at a 1:10 ratio, was added and incubated at room temperature for 30 min. Then, biotinylated antibody was added and incubated at 37 °C for 2 h. SA-HPR labeled secondary antibody (Sangon, Shanghai, China) was then added and incubated at 37 °C for 45 min. The reaction was developed by adding TMB substrate and stopped with 2 M sulfuric acid.

### 4.6. Neutralizing Assay

HIV-1 Env-pseudoviruses were generated by co-transfecting the pSG3∆env backbone and Env expression plasmids into 293T cells [46]. The serially diluted purified antibodies were incubated with the virus at 37 °C for 1 h. Then, the 1 × 10^4^ TZM-bl cells (NIH AIDS reagent program) in DMEM containing 10 µg/mL of DEAE-dextran were added into each well containing the virus and antibody. After 72 h of incubation, the Bright-Glo™ Luciferase substrate (Promega, Madison, WI, USA) was added into each well. The inhibition of the virus replication was determined by measuring relative light units (RLUs) on a the VICTOR2™ D fluorometer (PerkinElmer, Waltham, MA, Waltham, MA, USA). The 50% inhibitory concentrations (IC_50_) were defined as antibody concentrations at which RLUs were reduced by 50% compared with the average RLU of virus control. The heatmap was generated using the webtool at the Los Alamos HIV database (https://www.hiv.lanl.gov/content/sequence/HEATMAP/heatmap.html, accessed on 5 May 2023) [70].

### 4.7. Sequence Analysis

The amino acid sequences of the antibodies were compared using the sequence alignment tool (http://multalin.toulouse.inra.fr/multalin, accessed on 23 November 2023). In order to determine the evolutionary maturation of each antibody member in the same antibody lineage family, we aligned the antibody sequences of the same lineage and performed EMBL Simple Phylogeny analysis (https://www.ebi.ac.uk/Tools/phylogeny/simple_phylogeny, accessed on 24 November 2023). The iTOL tool was used for visualization analysis (https://itol.embl.de/upload.cgi, accessed on 24 November 2023).

### 4.8. Biolayer Interferometry Analysis

Biolayer interferometry (BLI) was used to detect the dynamic binding and dissociation between the antigen and the antibody. Briefly, 20 μg/mL JT18 or J038 antibodies were immobilized onto Anti-hIgG-Fc Capture (AHC) biosensors (FORTÉBIO, San Jose, CA, USA). The serially diluted BG505 UFO trimer was then added to the AHC biosensors. The binding was determined on the OCTET Red96 system (FORTÉBIO). Binding affinity constants were determined using OCTET software Data Analysis HT 9.0 (FORTÉBIO). To determine the binding affinity of V3 peptide to JT18, 447-52D, and J038 antibodies, the SHIV_1157ipd3N4_ V3 peptide (^305^KSISIGPGQAI^317^) was synthesized and its binding to the antibodies were determined as described above.

### 4.9. Site-Directed Mutagenesis

Designing forward and reverse primers centered on the mutated base site, and the SHIV_1157ipd3N4_ *env* gene was used as a template for mutation PCR. By using PCR, the primers can amplify the DNA segment containing the mutation or introduce a new mutation. To remove the original template, the PCR product was digested with Dpn I (Takara, Kusatsu, Shiga, Japan) at 37 °C overnight. Then, the digestion product was transformed into DH5*α* competent cells and the clones containing the right mutation(s) were screened by restriction enzyme digestion and confirmed by sequencing. The mutant pseudoviruses were generated by co-transfecting with the pSG3∆env backbone plasmid into 293T cells.

### 4.10. Statistical Analysis

All comparison analyses for the binding and neutralization data were performed with GraphPad version 8 (GraphPad Software, Inc., La Jolla, CA, USA). Data were presented with mean ± standard deviation. The *p*-value < 0.05 was considered significant. * indicates *p* < 0.05; **, *p* < 0.01; ***, *p* < 0.001; ns, not significant.

## Figures and Tables

**Table 1:** Determination of gene families and sequence characteristics of newly characterized antibodies.

	Heavy Chain					Light Chain			
ID	IGHV ^a^	IGHJ ^a^	IGHD ^a^	CDR3 ^b^ (aa)	SHM ^c^ (%)	IGKV/LV ^a^	IGKJ/LJ ^a^	CDR3 ^b^ (aa)	SHM ^c^ (%)
JT01	1-111*01	5-1*02	2-2*01	16	14.93	L1S1*01	L6*01	11	9.09
JT05	3-103*01	4*01	6-13*01	14	11.93	K1-25*02	K4*01	9	7.53
JT06	3-103*01	4*01	6-13*01	14	11.93	K1-25*01	K4*01	9	17.2
JT07	4-65*02	5-1*02	2-8*01	13	11.00	K1-32*04	K1*01	9	4.30
JT15	4-173*01	4*01	3-9*01	18	20.49	K1-25*01	K4*01	9	17.20
JT16	4-173*01	4*01	3-9*01	18	18.06	K1-25*01	K4*01	9	20.79
JT18	5-20*02	5-2*02	1-44*02	12	12.85	L1S1*01	L3*01	11	7.61
JT19	5-43*01	5-1*01	1-44*01	16	13.54	L3-22*01	L3*01	11	10.71

^a^ The mAb sequences were assigned to the closest known macaque germline V genes, D genes, and J genes, as appropriate. ^b^ The mAb CDR3 lengths were determined according to the Kabat definition. ^c^ The SHM (%) were obtained by calculating the proportion of differences between the antibody and the corresponding germline antibody.

**Table 2:** Half maximal effective concentration (EC_50_) of newly characterized mAbs.

EC_50_ (μg/mL)	JT01	JT05	JT06	JT07	JT15	JT16	JT18	JT19
SHIV_1157ipd3N4_ gp120 Monomer	0.0072	0.0850	196.0	94.82	0.0272	0.0209	0.0050	99.88
BG505 UFO trimer	0.0098	133.5	205.4	48.34	0.0893	0.1077	0.0212	107.4

## Data Availability

Data are contained within the article. The original data presented in the study are openly available in FigShare at https://doi.org/10.6084/m9.figshare.25857379.

## Supplementary Materials

The following supporting information can be downloaded at: https://www.mdpi.com/article/10.3390/ijms25137200/s1.
