# Supplementary material for: Recapitulation of HIV-1 Neutralization Breadth in Plasma by the Combination of Two Broadly Neutralizing Antibodies from Different Lineages in the Same SHIV-Infected Rhesus Macaque
Source: Int J Mol Sci. 2024 Jun 29;25(13):7200. doi: 10.3390/ijms25137200 (PMC11240982; doi:10.3390/ijms25137200)
Supplement: Supplementary file 1 [file ijms-25-07200-s001.zip › Figures S1 and S2.pptx]

## Slide 1
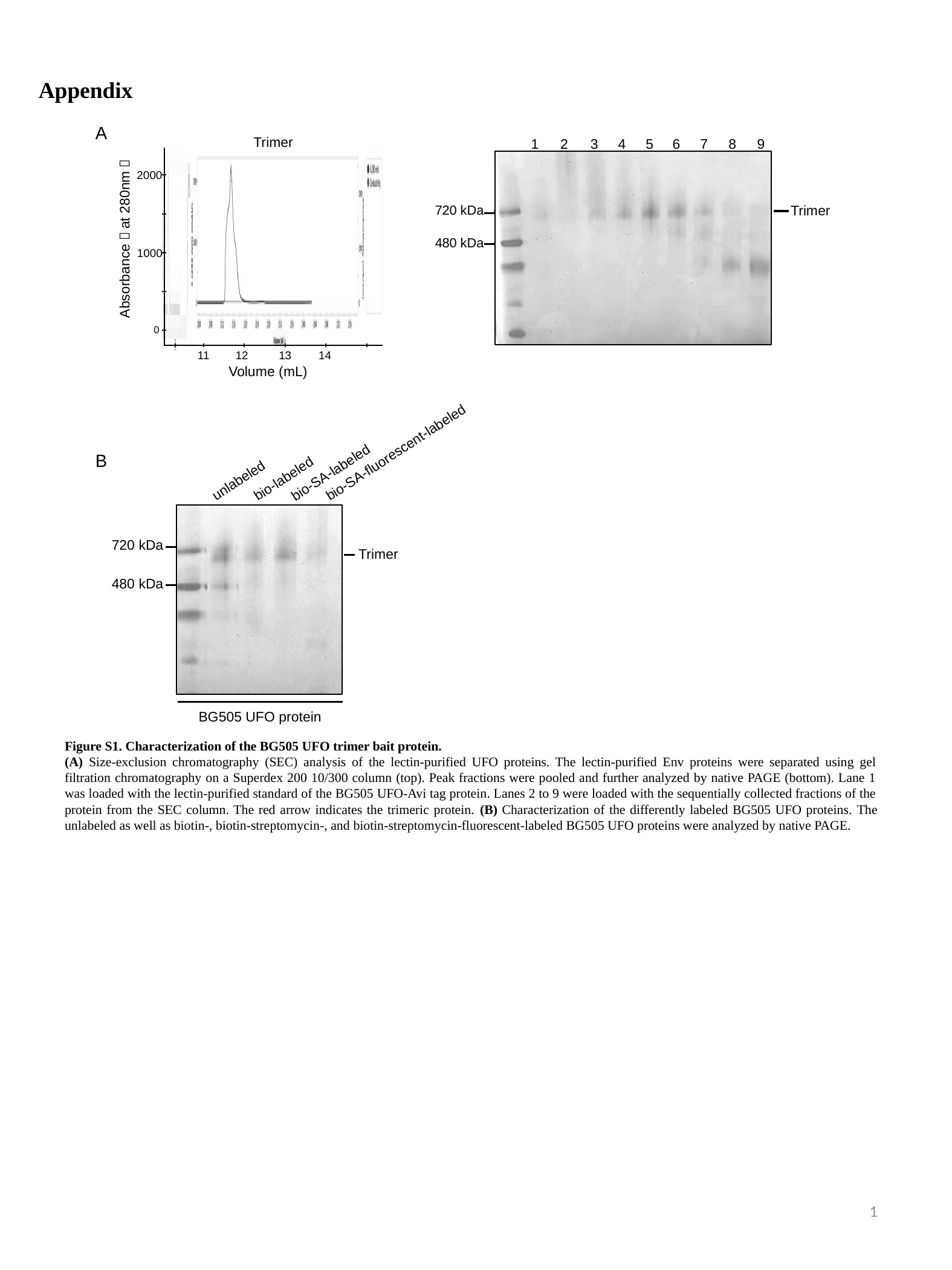

Appendix
A
Trimer
1
2
3
4
5
6
7
8
9
2000
Absorbance（at 280nm）
1000
0
Trimer
720 kDa
480 kDa
11
12
13
14
Volume (mL)
bio-SA-fluorescent-labeled
bio-SA-labeled
B
unlabeled
bio-labeled
720 kDa
Trimer
480 kDa
BG505 UFO protein
Figure S1. Characterization of the BG505 UFO trimer bait protein.
(A) Size-exclusion chromatography (SEC) analysis of the lectin-purified UFO proteins. The lectin-purified Env proteins were separated using gel filtration chromatography on a Superdex 200 10/300 column (top). Peak fractions were pooled and further analyzed by native PAGE (bottom). Lane 1 was loaded with the lectin-purified standard of the BG505 UFO-Avi tag protein. Lanes 2 to 9 were loaded with the sequentially collected fractions of the protein from the SEC column. The red arrow indicates the trimeric protein. (B) Characterization of the differently labeled BG505 UFO proteins. The unlabeled as well as biotin-, biotin-streptomycin-, and biotin-streptomycin-fluorescent-labeled BG505 UFO proteins were analyzed by native PAGE.
1

## Slide 2
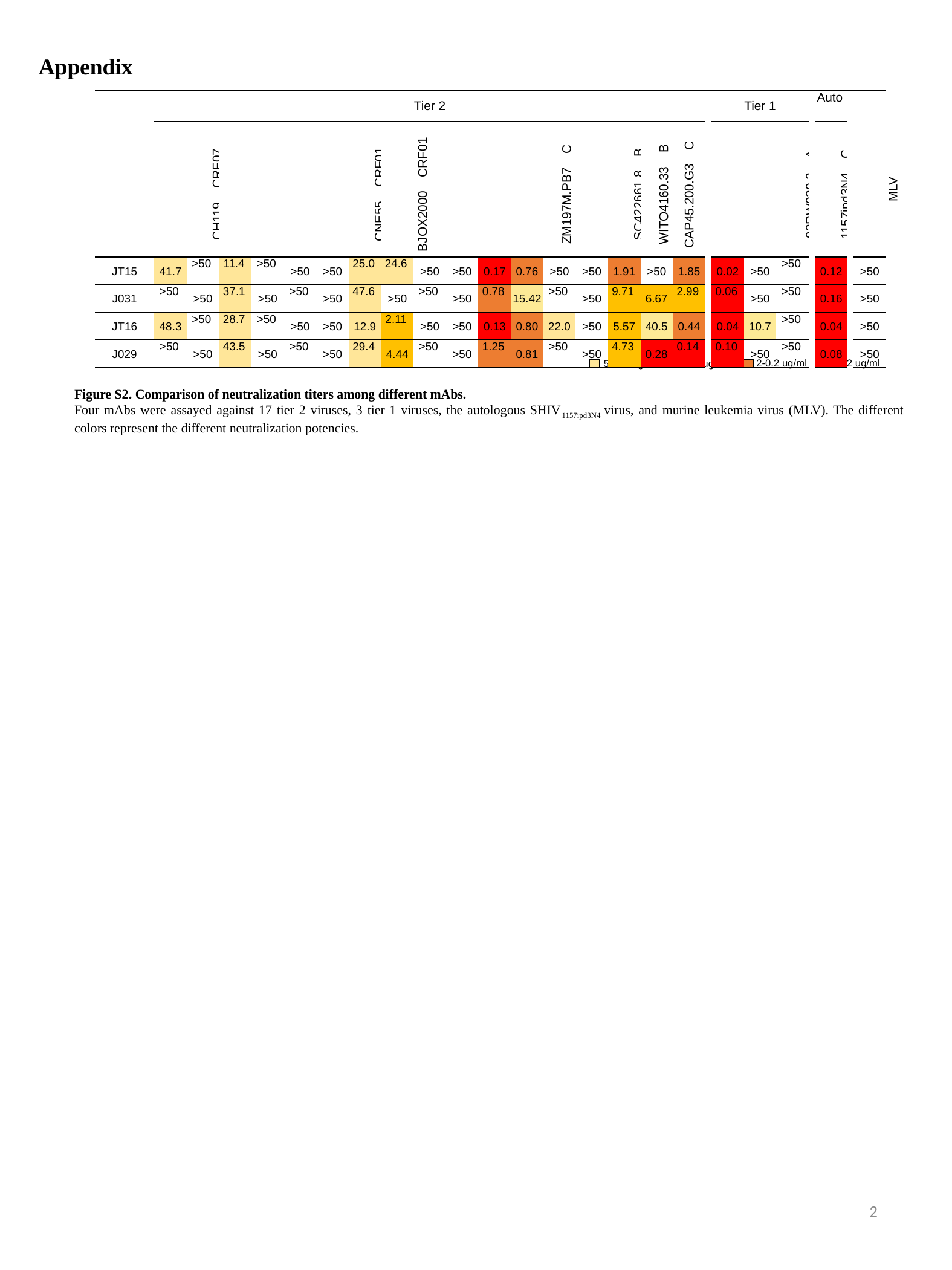

Appendix
| | Tier 2 | | | | | | | | | | | | | | | | | | Tier 1 | | | | Auto | | |
| --- | --- | --- | --- | --- | --- | --- | --- | --- | --- | --- | --- | --- | --- | --- | --- | --- | --- | --- | --- | --- | --- | --- | --- | --- | --- |
| | X1632（G） | CH119（CRF07） | TRO11（B） | 246F3（AC） | 398F1（A） | CNE8（CRF01） | CNE55（CRF01） | CE0217（C） | BJOX2000（CRF01） | CE1176（C） | X2278（B） | 25710（C） | ZM197M.PB7（C） | 16936-2.21（C） | SC422661.8（B） | WITO4160.33（B） | CAP45.200.G3（C） | | MW965.26 （C） | SF162.LS（B） | 92RW020.2（A） | | 1157ipd3N4（C） | | MLV |
| JT15 | 41.7 | >50 | 11.4 | >50 | >50 | >50 | 25.0 | 24.6 | >50 | >50 | 0.17 | 0.76 | >50 | >50 | 1.91 | >50 | 1.85 | | 0.02 | >50 | >50 | | 0.12 | | >50 |
| J031 | >50 | >50 | 37.1 | >50 | >50 | >50 | 47.6 | >50 | >50 | >50 | 0.78 | 15.42 | >50 | >50 | 9.71 | 6.67 | 2.99 | | 0.06 | >50 | >50 | | 0.16 | | >50 |
| JT16 | 48.3 | >50 | 28.7 | >50 | >50 | >50 | 12.9 | 2.11 | >50 | >50 | 0.13 | 0.80 | 22.0 | >50 | 5.57 | 40.5 | 0.44 | | 0.04 | 10.7 | >50 | | 0.04 | | >50 |
| J029 | >50 | >50 | 43.5 | >50 | >50 | >50 | 29.4 | 4.44 | >50 | >50 | 1.25 | 0.81 | >50 | >50 | 4.73 | 0.28 | 0.14 | | 0.10 | >50 | >50 | | 0.08 | | >50 |
2-0.2 ug/ml
<0.2 ug/ml
50-10 ug/ml
10-2 ug/ml
Figure S2. Comparison of neutralization titers among different mAbs.
Four mAbs were assayed against 17 tier 2 viruses, 3 tier 1 viruses, the autologous SHIV1157ipd3N4 virus, and murine leukemia virus (MLV). The different colors represent the different neutralization potencies.
2

## Slide 3
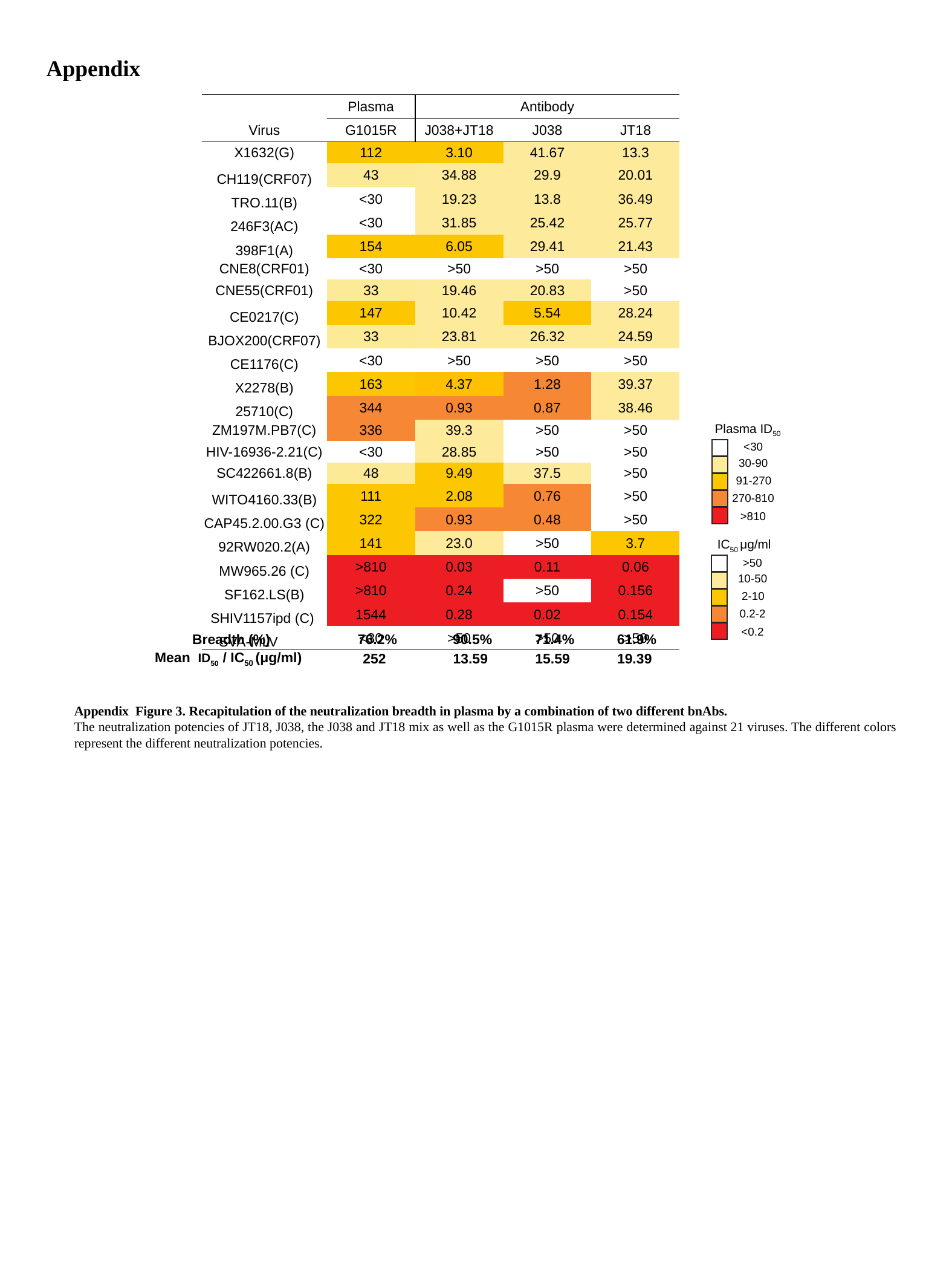

Appendix
| | Plasma | Antibody | | |
| --- | --- | --- | --- | --- |
| Virus | G1015R | J038+JT18 | J038 | JT18 |
| X1632(G) | 112 | 3.10 | 41.67 | 13.3 |
| CH119(CRF07) | 43 | 34.88 | 29.9 | 20.01 |
| TRO.11(B) | <30 | 19.23 | 13.8 | 36.49 |
| 246F3(AC) | <30 | 31.85 | 25.42 | 25.77 |
| 398F1(A) | 154 | 6.05 | 29.41 | 21.43 |
| CNE8(CRF01) | <30 | >50 | >50 | >50 |
| CNE55(CRF01) | 33 | 19.46 | 20.83 | >50 |
| CE0217(C) | 147 | 10.42 | 5.54 | 28.24 |
| BJOX200(CRF07) | 33 | 23.81 | 26.32 | 24.59 |
| CE1176(C) | <30 | >50 | >50 | >50 |
| X2278(B) | 163 | 4.37 | 1.28 | 39.37 |
| 25710(C) | 344 | 0.93 | 0.87 | 38.46 |
| ZM197M.PB7(C) | 336 | 39.3 | >50 | >50 |
| HIV-16936-2.21(C) | <30 | 28.85 | >50 | >50 |
| SC422661.8(B) | 48 | 9.49 | 37.5 | >50 |
| WITO4160.33(B) | 111 | 2.08 | 0.76 | >50 |
| CAP45.2.00.G3 (C) | 322 | 0.93 | 0.48 | >50 |
| 92RW020.2(A) | 141 | 23.0 | >50 | 3.7 |
| MW965.26 (C) | >810 | 0.03 | 0.11 | 0.06 |
| SF162.LS(B) | >810 | 0.24 | >50 | 0.156 |
| SHIV1157ipd (C) | 1544 | 0.28 | 0.02 | 0.154 |
| SVA-MLV | <30 | >50 | >50 | >50 |
Plasma ID50
<30
30-90
91-270
270-810
>810
IC50 μg/ml
>50
10-50
2-10
0.2-2
<0.2
| Breadth (%) | 76.2% | 90.5% | 71.4% | 61.9% |
| --- | --- | --- | --- | --- |
| Mean ID50 / IC50 (μg/ml) | 252 | 13.59 | 15.59 | 19.39 |
| --- | --- | --- | --- | --- |
Appendix Figure 3. Recapitulation of the neutralization breadth in plasma by a combination of two different bnAbs.
The neutralization potencies of JT18, J038, the J038 and JT18 mix as well as the G1015R plasma were determined against 21 viruses. The different colors represent the different neutralization potencies.
